# Recent Progress in the Development of Graphene Detector for Terahertz Detection

**DOI:** 10.3390/s21154987

**Published:** 2021-07-22

**Authors:** Jianlong Liu, Xin Li, Ruirui Jiang, Kaiqiang Yang, Jing Zhao, Sayed Ali Khan, Jiancheng He, Peizhong Liu, Jinfeng Zhu, Baoqing Zeng

**Affiliations:** 1 National Key Laboratory of Science and Technology on Vacuum Electronics, School of Electronic Science and Engineering, University of Electronic Science and Technology of China, Chengdu 610054, China; liujianlong@uestc.edu.cn (J.L.); 201922021527@std.uestc.edu.cn (X.L.); 201711040120@std.uestc.edu.cn (R.J.); 201811022515@std.uestc.edu.cn (K.Y.); 202011022910@std.uestc.edu.cn (J.Z.); 201921020407@std.uestc.edu.cn (J.H.); bqzeng@uestc.edu.cn (B.Z.); 2 Institute of Electromagnetics and Acoustics, School of Electronic Science and Engineering, Xiamen University, Xiamen 361005, China; sayedali@mail.ustc.edu.cn; 3 Department of the Internet of Things Engineering, College of Engineering, Huaqiao University, Quanzhou 362000, China; pzliu@hqu.edu.cn

**Keywords:** graphene, terahertz detector, photovoltaic effect, photothermoelectric effect, plasma wave resonance

## Abstract

Terahertz waves are expected to be used in next-generation communications, detection, and other fields due to their unique characteristics. As a basic part of the terahertz application system, the terahertz detector plays a key role in terahertz technology. Due to the two-dimensional structure, graphene has unique characteristics features, such as exceptionally high electron mobility, zero band-gap, and frequency-independent spectral absorption, particularly in the terahertz region, making it a suitable material for terahertz detectors. In this review, the recent progress of graphene terahertz detectors related to photovoltaic effect (PV), photothermoelectric effect (PTE), bolometric effect, and plasma wave resonance are introduced and discussed.

## 1. Introduction

The terahertz (THz) wave is an electromagnetic wave in a particular band having a frequency range of 0.1–10 THz (wavelength within 30–3000 microns) [1]. This frequency band is within the electronics-to-photonics transition region, and it leads the THz waves to have certain characteristics of the microwaves and the light waves. Comparing with the microwaves and millimeter waves, the THz imaging system is capable of a higher resolution in detection and imaging [2]. More interestingly, THz waves penetrate dust and smoke more easily than infrared and visible light, making them ideal for working in harsh environments [3,4,5]. Furthermore, THz waves often have a strong capacity to penetrate non-polar materials, such as leather and plastics, and they are capable of effectively detecting concealed hazardous items, such as explosives, drugs, and firearms along with other prohibited objects, which make the THz waves important for applications in security [6,7,8]. The THz waves are also suitable for biomedical imaging and medical diagnosis of various diseases due to their low energy photons compared to X-rays, which do not cause any adverse effects of photoionization on biological tissues [9,10].

**Figure 1:**
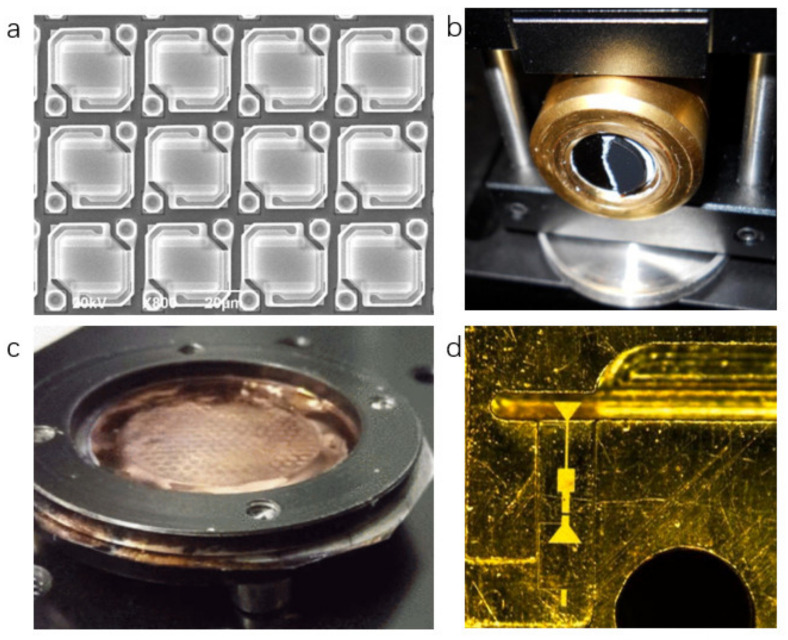
(**a**) The image of Bolometer array. Reprinted with permission from ref. [33]. Copyright 1969 Elsevier. (**b**) Appearance of pyroelectric detector. Reprinted with permission from ref. [34]. Copyright 2021 Copyright Clearance Center. (**c**) Appearance of Golay cell. Reprinted with permission from ref. [35]. Copyright 2021 Copyright Clearance Center. (**d**) Photograph of Schottky diode. Reprinted with permission from ref. [36]. Copyright 2021 Copyright Clearance Center.

The THz detectors mainly include bolometer, pyroelectric detector, Golay cell, Schottky diode detector, nanometric field-effect transistor (NFET), etc. [11]. The bolometer relies on temperature-related resistance. When the THz wave radiates to the working area, the resistance of the thermistor will change. This change can reflect the radiation intensity of terahertz [12,13,14]. This kind of detector usually works at low temperatures, which needs to be equipped with refrigeration equipment [15]. The pyroelectric detector operates via the pyroelectric effect of the crystal. Owing to the absorption of THz waves, the temperature of the crystal increases and cause a change of carrier concentration. A potential difference is formed at both ends of the crystal, and the energy of the THz wave can be calculated according to the magnitude of the potential difference, and the potential difference will disappear when the internal temperature of the crystal is balanced [16,17,18]. The working principle of the Golay cell detector is that the gas absorbs energy and expands as the THz wave irradiates the gas cavity, causing deformation of the film in the cavity, and the identification of the THz wave can be accomplished by calculating the deformity [19]. The Schottky diode detector is made of a Schottky diode between the metal and semiconductor. This kind of detector usually working at room temperature (RT) [20,21,22]. The NFET mainly uses the plasma wave excited in the channel of the device to detect the THz wave. Since the excitation of the plasma wave requires only a small amount of energy, this detector has high responsiveness [23]. The device is small in size and easy to integrate [24]. In addition, there is research on using bow-tie diodes made of GaAs or InGaAs to detect THz waves [25,26,27]. The working parameters of these detectors are shown in Table 1, and the digital photographs of these detectors are depicted in Figure 1.

**Table 1:** Performance parameters of terahertz detectors.

Type	Response Time	Frequency Range	Noise-Equivalent Power (NEP)	Reference
Bolometer	10 µs	-	$10^{-9}\;W/{\mathrm{Hz}}^{1/2}$	[20,28]
Pyroelectric detector	less than 10 ms	0.1–30 THz	$10^{-10}\;W/{\mathrm{Hz}}^{1/2}$	[17,29]
Golay cell	10 ms	0.1–20 THz	$10^{-10}\;W/{\mathrm{Hz}}^{1/2}$	[30]
Schottky diode	100 ps	0.1–1.7 THz	$10^{-10}\;W/{\mathrm{Hz}}^{1/2}$	[20,22]
NFET	less than 30 ps	0.3–4.9 THz	$10^{-12}\;W/{\mathrm{Hz}}^{1/2}$	[24,31,32]
Bow-tie diode	7 ns	0.1–2.5 THz	$10^{-9}\;W/{\mathrm{Hz}}^{1/2}$	[25,26,27]

The nature of the materials and their structures have a significant impact on the performance THz detector [37]. For example, the mobility of the materials used in the THz detector has a direct impact on their sensitivity. More specifically, when the material mobility is high enough, the device can induce resonant surface plasma oscillations in the channel, which can dramatically reduce the noise equivalence power (NEP). With the advancement of material, two-dimensional (2D) materials with excellent photoelectric properties have been produced and applied to THz detectors such as graphene, black phosphorus, and transition metal dichalcogenides [38,39].

It had been Geim and Novoselov, who used mechanical exfoliation to develop graphene, a single-atomic-layer graphite structure [40]. Graphene is a 2D material with a hexagonal honeycomb lattice composed of carbon atoms and sp2 hybrid orbitals [41]. The arrangement of carbon atoms in graphene determines its unique conical band structure, which makes graphene have some unique properties, including a quantum Hall effect at room temperature, adjustable conductivity, small carrier equivalent mass, very large mean free path, etc. [42,43]. Moreover, graphene has a theoretical value of intrinsic electron mobility up to 200,000 ${\mathrm{cm}}^{2}/V\cdot s$ at room temperature, which is 140 times greater than silicon. In addition, a stable atomic structure with only weak van der Waals forces at the interface makes it favorable for other materials to form a heterostructure [42,44,45]. The graphene has great application potential in various fields such as NFET, nano-electronic devices, high-sensitivity sensors, etc. [46,47,48]. The 2D planar structure composed of the atomic layers can be regarded as the thinnest two-dimensional electron gas. The high carrier mobility, adjustable conductivity, and other promising characteristics make graphene a novel material for far-infrared radiation (FIR) and THz devices, especially novel photodetectors [49].

## 2. Classification of Graphene Terahertz Detectors

The most commonly used THz detectors have some limitations for specific applications. For example, the response speed of the pyroelectric detectors and Golay cell is low, and it is difficult to respond in real time to ultra-short THz pulse signals in the order of picoseconds [17,50]. Schottky diode detectors can only have good responsivity below 1 THz due to their parasitic parameters but have lower damage threshold energy, which makes it difficult to resist the powerful electric field of terahertz irradiation and easy to be damaged in practical applications. Graphene has a unique zero bandgap structure with exceptionally high carrier mobility and can be modified for conductivity; those excellent features make it an ideal material for making THz detectors [51,52,53,54]. The THz detectors fabricated with graphene boost the working frequency of the device by efficiently reducing the impact of major noise sources of the high-frequency range transistor, effectively lowering the noise of the high-frequency devices. The working principle of a graphene detector mainly includes the photovoltaic effect (PV), photoconductivity effect, photo-thermoelectric effect (PTE), bolometer effect, plasma wave resonance, etc. Among these working principles, some of them change the conductivity after absorbing radiation, and a bias voltage is needed to detect this change. Others generate photocurrent by themselves after absorbing radiation, and this kind of detector does not require a bias voltage. According to this characteristic, we divide graphene THz detectors into two categories.

### 2.1. Non-Zero Bias Detectors

**Figure 2:**
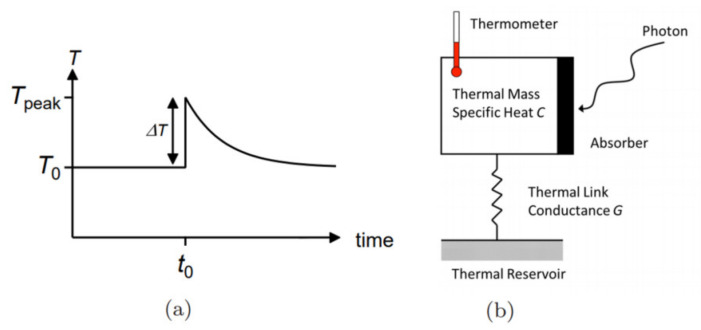
(**a**) The temperature response T (t) for a calorimeter. (**b**) Schematic illustration of bolometer working principle in a graphene detector, C (heat capacity) and the thermometer function are provided by the electron subsystem. G (thermal conductance) is provided by the coupling to the contacts and substrate as well as emitted Johnson noise. Reprinted with permission from ref. [56]. Copyright 2021 Copyright Clearance Center.

In general, most of the graphene non-zero bias detectors are graphene bolometers. A bolometer is a device that uses the bolometer effect to detect the intensity of the radiation power. The bolometer is based on an electrical resistance’s temperature dependency. More specifically, the working mechanism of the bolometer is to convert absorbed terahertz radiation energy to a changing temperature value by absorbing a photon and increasing the electron temperature from $T_{0}$ to $T_{\mathrm{peak}}$, as illustrated in Figure 2a, causing a change in the thermosensitive resistance of materials. To measure terahertz radiation, a bias voltage needs to be added to the device, and the current increases linearly with the voltage. The thermal resistance and the heat capacity are the key parameters of the device, as illustrated in Figure 2b [55,56]. Notably, the electron heat capacity in graphene is small, and the electron–phonon coupling is weak [57,58]. These advantages are very suitable for making a thermal detector with fast response time, higher sensitivity, and less noise.

Ryzhii et al. [59] report a bolometer theoretical model with bilayer graphene. One layer of graphene is doped n-type, and the other layer is doped p-type. Both of them are connected by an array of undoped graphene nano-ribbons (GNRs). The graphene layer absorbs THz radiation, and the GNR array acts as a barrier area. The computational analysis validates that the voltage response of this structure’s bolometer can reach $1\times 10^{5}\;V/W$, indicating that it has superior performance parameters compared to the bolometers discussed previously [28]. This work provides a theoretical basis for the fabrication of a graphene bolometer.

Yan et al. [57] designed a bolometer that uses 2D graphene bulk instead of a one-dimensional rectifying interface. The poor interaction between electrons and phonons within the graphene would create a bottleneck in the thermal direction of decoupling electrons thermally from the photonic bath after absorbing THz radiation. They used the non-linear nature of the light response to measure the response speed of the bolometer under different laser sources. The response curve is consistent with the bolometric model. The time average signal of the detector is determined by the slowest scattering process, while the fast scattering process does not contribute much. They considered thermal noise and Johnson–Nyquist noise when measuring progressive NEP; the response time is 1.2 ms at 4.2 K temperature, and NEP is as low as $1.2\times 10^{-13}\;W/{\mathrm{Hz}}^{1/2}$. Under the assumption of a $T^{-3}$ dependence of thermal resistance $R_{h}$ and a linear T dependence of the specific heat, the detector can have better performance at a lower temperature ($1.5\times 10^{-20}\;W/{\mathrm{Hz}}^{1/2}$ at 100 mK with a response time of 3 µs). Reducing the volume of the detector can reduce the heat capacity and improve the sensitivity, which suggested that there is a big improvement needed in the future.

The conventional approach of using electrical transport as a readout detector can hardly realize the supervisory characteristic of graphene for making high-sensitivity THz detectors. A thermionic electron detector based on noise readout also has been reported [60]. The temperature of the thermionic electrons is determined by monitoring the thermal noise power of the detector as well as the temperature change caused by the incident wave. In the state of thermal equilibrium, the electron follows the Fermi–Dirac distribution in the quantum state. Electron absorption will more aggressively absorb energy since the temperature increases, and the possibility of energy levels being occupied by electrons above the Fermi level will increase. The absorbed THz radiation can cause the temperature change of electrons in the material; then, electron transfer and energy band change occurred [61,62,63,64].

With very weak coupling and high-speed thermal response, graphene could act as a highly sensitive bolometer. Fong et al. [65] introduced microwave frequency noise thermometry to study these delicate and wide-bandwidth thermal features. The measurement on the graphene sample is to provide a thermal conductance channel for cooling through the emission of photons into the measurement channel. This Johnson noise-based method can reach the thermodynamic limit. The measurement results are related to the theory of the electron–phonon coupling, which shows that the measurement of THz wave with noise in a low-temperature environment is a relatively ideal method. They used the fabricated bolometer to realize the heat capacity of a two-dimensional electron gas at zero fields.

**Figure 3:**
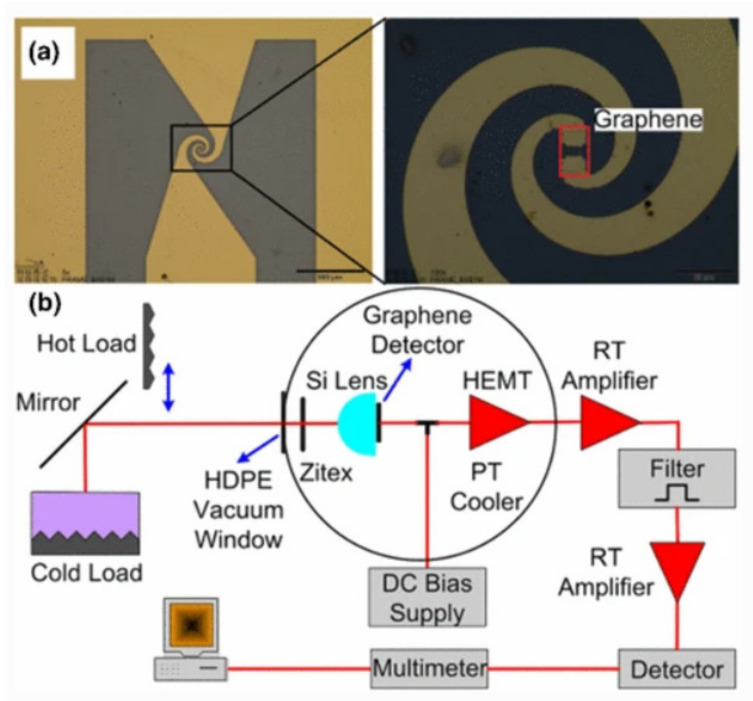
(**a**) Image of the graphene-based thermal noise readout bolometer. (**b**) The schematic illustration of the measurement system. Reprinted with permission from ref. [66]. Copyright 2021 Copyright Clearance Center.

Miao et al. [66] reported a graphene-based thermal noise readout bolometer. The detector is composed of graphene and a THz spiral metal antenna. The bolometer is a graphene micro-bridge connected to the logarithmic spiral antenna through Au contact. The detector had a THz response with the band range from 0.3 to 1.6 THz, and the NEP is $5.6\times 10^{-12}\;W/{\mathrm{Hz}}^{1/2}$ at a temperature of 3 K. Figure 3a shows the structure, and Figure 3b is the system test diagram of the detector. In order to understand the heat transfer of graphene microbridges, they measured three kinds of microbridge length (from 0.6 to 8 μm) and measure the detectors at different temperatures. The results illustrate that the length of the graphene microbridge has little influence on the NEP, but it is largely affected by the heat diffusion of the contact pad because of the electron diffusion. Using superconducting contact pads can improve the performance of the bolometer.

### 2.2. Zero-Bias Detectors

In graphene zero-bias detectors, a built-in electric field is formed inside the material after absorbing THz radiation, so it can realize zero-bias detection. The built-in electric field can be enhanced by local mixing or the use of contact asymmetry. The working principle of graphene zero-bias detector mainly includes a photovoltaic effect, photo-thermoelectric effect, plasma wave resonance, etc. [67,68]. Compared with conventional photodetectors, such as GaAs/AlGaAs devices, graphene-based detectors have a wider detection wave range and faster response speed [69]. In theory, an antenna-coupled THz detector made by graphene with mobility of 1500${\;\mathrm{cm}}^{2}/V\cdot s$ can achieve A/W-level current responsivity and the NEP below $50\;\mathrm{pW}/{\mathrm{Hz}}^{1/2}$ [70]. According to the different dominant mechanisms of photocurrent generation, we can divide these zero-bias detectors into two categories: one is the detector with PTE or PV, and the other is the detector with plasma wave resonance.

#### 2.2.1. The Detector Based on Photovoltaic Effect or Photothermoelectric Effect

The detection mechanism of the photovoltaic effect means that when electromagnetic waves are irradiated on the detector, the electrons in the valence band of the photoelectric material absorb the energy according to the equation of Ε=hυ (h is Planck constant and υ is frequency) of the photon, which leads to excite an electron to the conduction band of the high-energy state and leave a hole in the valence band of the low-energy state. The photogenerated electron–hole pairs are separated by the built-in electric field and form a photogenerated electric field opposite to the built-in electric field. The mechanism of the photothermoelectric effect is that when the photothermal material absorbs photons, the internal lattice vibration accelerated, and the temperature rises. The heat makes a part of the detector higher than the ambient temperature, which can make a temperature gradient inside the material. As a result, carrier concentrations between the hot and cold ends are different, which drives the multi-carriers in the hot end of the material to move to the cold end of the material and form a thermoelectric voltage $V_{\mathrm{PTE}}=(S_{1}-S_{2})\Delta T$, where ΔT is temperature difference and S are Seebeck coefficients that are related to the conductivity. Fast photoelectric conversion can be realized due to the ultra-fast change of graphene conductivity with the radiation.

**Figure 4:**
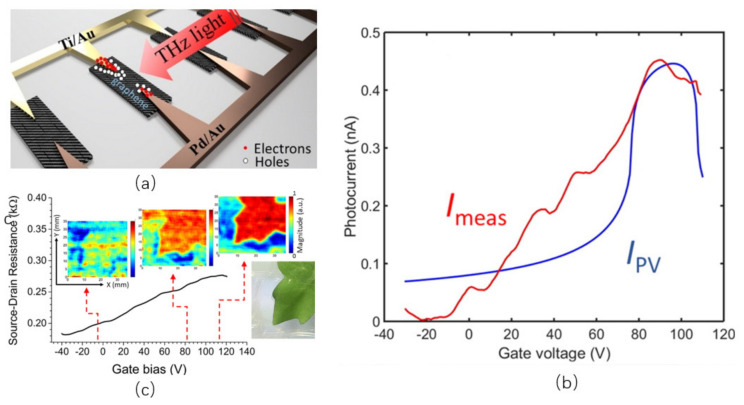
(**a**) Illustration of the photovoltaic-based detector concept map. (**b**) Blue trace $I_{\mathrm{PV}}$ is the calculated photovoltaic current, and red trace $I_{\mathrm{meas}}$ is the measured photocurrent. (**c**) The THz diagrams of fresh blades under different grid bias voltages. The three photos correspond to the bias voltages of 0, 80, and 110 V respectively. Reprinted with permission from ref. [73]. Copyright 2016 American Chemical Society.

The photodetector usually combines the effect of the PV and the PTE, but their role during the detection can be far different [71,72]. Degl et al. [73] reported a THz detector with a graphene coupled antenna which is based on the photovoltaic effect. In order to improve the responsiveness and coupling of incident light to the detector, they used two different metals to make an antenna array. As illustrated in Figure 4a, one side of the antenna is built of Ti/Au to achieve n-type doping in nearby graphene, while the other is made of Pd/Au to achieve p-type doping. As a result of the photovoltaic effect, they built an electric field at a metal–graphene junction, which separates the photo-excited electron-hole pairs. There are multiple changes in the direction of the photocurrent generated by PTE in graphene because the Fermi energy is varied along the Dirac cone. The photocurrent in different back-gate voltages cannot be reversed, as shown in Figure 4b, which suggests that the photovoltaic effect dominates the detector. They also integrate the detector into the imaging system and receive THz imaging of fresh leaves, as shown in Figure 4c.

**Figure 5:**
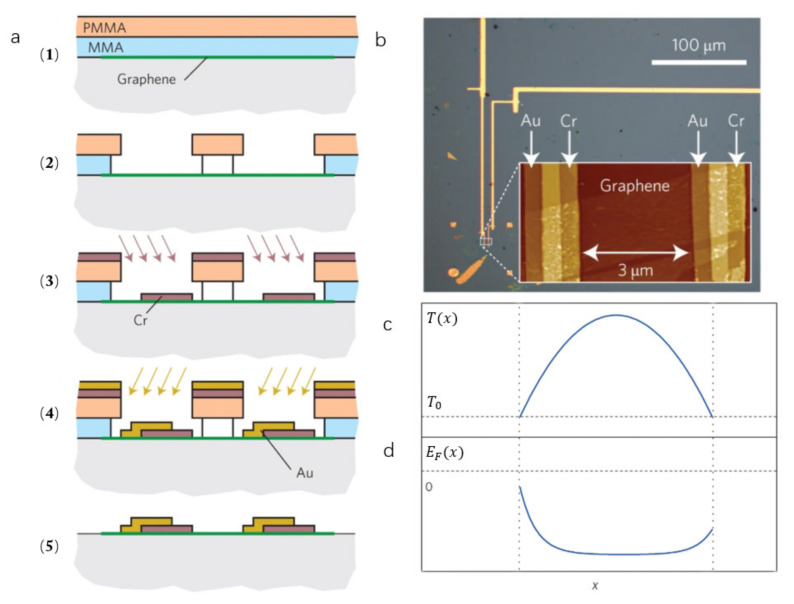
(**a**) Schematic illustration of the manufacturing mechanism of the detector with double angle evaporation technology. (1) MMA/PMMA is spun onto graphene. (2) Resist is patterned by an electron beam and developed. Successive angled evaporations of chromium (3) and gold (4) followed by liftoff produces a single-layer graphene device with dissimilar metal contacts on the opposing sides as shown schematically in (5). (**b**) The image of the electrical connection point of the detector in an optical microscope. (**c**,**d**) The curve of temperature (T (x)) and Fermi level ($E_{F\;}$ (x)) at different positions in graphene. Reprinted with permission from ref. [74]. Copyright 2021 Copyright Clearance Center.

Temperature-dependent resistance in bilayer graphene is important in graphene detectors that use the photothermoelectric effect and must work at low temperatures [57,65]. Cai et al. [74] reported a THz detector based on the photothermoelectric effect. More interestingly, they generated an asymmetry by contacting graphene with dissimilar metals using a standard double-angle evaporation technique, as shown in Figure 5a. The electrons in graphene are heated by absorbing the energy of the THz wave. They found that the Fermi energy profiles are changed in the different devices due to their different metal contacts, and the heat dissipation of the contacts makes the temperature distribution of the device different, as shown in Figure 5c,d. By using the absorbed power instead of the incident power to define the response, the measurement allows for a better comparison of responses at different wavelengths. Interestingly, the sensitivity of detector is better than 10 V/W without a coupling antenna, indicating that the performance of the THz detector has great potential for improvement.

The structure of the coupling antenna for the PTE THz detector can also considerably improve the detector’s performance. Guo et al. [75] used ultraviolet lithography to prepare a square metal spiral antenna. This structure of antenna can increase the absorption area, approximately stabilize radiation impedance in the THz band, and be widely used in broadband detection [76]. For THz wave coupling and metallic electrodes, the antenna can be employed. Graphene is laid on the right side and connected to the metal in three places, which can ensure asymmetry without using different metal contacts. The maximum response of the detector is 28 V/W, and the response time is 9 µs. Compared with the detectors without an antenna [74], the detector coupled with the antenna has better performance in response and NEP. The electron heat capacity of graphene is low; thus, the temperature fluctuates dramatically under the same absorption energy, resulting in a higher photocurrent generated by the detector and increased sensitivity. For graphene, the carriers are heated by electron–electron interaction, which is faster than the electron-phonon method. Moreover, the heat balance maintains for a long time in graphene; as a result, the response speed of the graphene detector is fastened.

**Figure 6:**
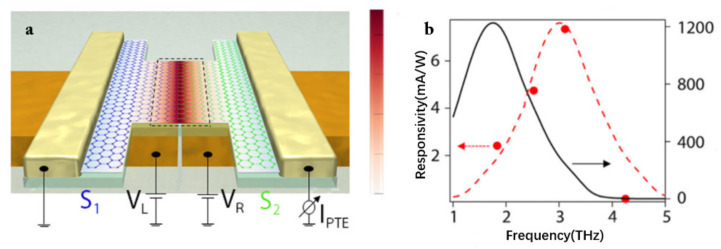
(**a**) The schematic illustration of the fabricated device. Appropriate voltage is applied to both sides of the antenna, and the terahertz radiation will be concentrated in the central part of the graphene channel (where incident terahertz wave is concentrated by the antenna). (**b**) The black line is the simulation result, and the red line is the trend of the experimental results. Reprinted with permission from ref. [72]. Copyright 2019 American Chemical Society.

Another significant parameter to improve the characteristic of the THz detector is reducing the device resistance [77]. In 2019, Castilla et al. presented a sort of THz detector [78] that is connected with a two-branched antenna with a gap of roughly 100 nm between the antennas, as illustrated in Figure 6a. By applying an appropriate voltage on both the branches of the antenna, a p–n junction in the graphene channel is created through the capacitive coupling, which had a small antenna gap and makes the carriers have a higher temperature under enhanced radiation intensity. The THz radiation is concentrated and enhanced in this p-n junction, resulting in a photocurrent response. There is no electrical connection between the detector antenna and the graphene. Meanwhile, the resistance of the entire device is reduced, and the performance of the detector is remarkably enhanced. By measuring the photocurrent as a function of the bias voltage applied between the source and drain contacts, it is found that the PTE is the main mechanism that dominates the photon response of the detector. In addition, the incident wave frequency is changed from 1.83 to 4.25 THz, and the response center is observed at about 3 THz. However, the response center is at 2 THz in the full-wave simulation, as shown in Figure 6b. This discrepancy can be attributable to the antenna’s limitations. Using an antenna with a wider spectral range will expand the detection range of the detector.

In addition, there are many studies on the performance of THz detectors. Metamaterials are an effective way to enhance the performance of graphene photodetectors [78,79]. The absorption of THz can be greatly enhanced by a periodically tunable plasma perfect metamaterial absorber with a square–square–circle graphene array [80]. A large area and rapid THz detector was accomplished by transferring graphene on a patterned silicon substrate to establish a Schottky junction [81]. Using three-dimensional graphene foam could also make ultra-wideband and high-sensitivity THz detectors [82]. Other researchers have studied the physical properties of graphene carrier transport and terahertz-thermoelectric conversion under different annealing treatments [83].

#### 2.2.2. The Photodetector Based on Plasma Wave Resonance

In 1993, Dyakonov and Shur proved that the electron behavior in a short channel field effect transistor (FET) is similar to that in shallow water, which provides a new mechanism to detect THz [84,85]. The plasmonic wave is the basic mechanism of the graphene field effect transistor (GFET) utilized as a THz detector. When there is a high concentration of electrons in the channel of the FET, the electrons will collide during the transmission and do not follow the ballistic transmission mechanism. In this case, the electrons can be regarded as two-dimensional electron gas [84,86,87]. When the THz wave is irradiated on the detector, it will excite the plasmonic wave. The channel of the device can just become the resonant cavity of the plasmonic wave. With the resonance, a voltage or current will be generated between the source and the drain. The magnitude of the generated current or voltage is related to the power of the incident wave [68,88]. In FET, the frequencies of the eigen plasmon modes are

(1) $$\omega_{N}\;=\;\left(2N-1\right)\omega_{0}$$

(2) $$\omega_{0}=\frac{\pi }{2L}\sqrt{\frac{e\left(U_{g}-U_{\mathrm{th}}\right)}{m^{*}}}$$

where L is the gate length, $U_{g}$ is the gate voltage, $U_{\mathrm{th}}$ is the threshold voltage, and e and $m^{*}$ are the electron charge and effective mass, respectively. The resonance mode can be adjusted by adjusting the size of the channel or the electron density [89], the concentration of carriers in graphene could be tuned by doping. When the carrier concentration is moderate, the plasmonic oscillation frequency in graphene is in the THz band [90,91]. A broadband THz detector can be made by modulating the Fermi energy level and changing the structure size [92]. The electrons in graphene are massless Dirac fermions [93], so the coupling of electromagnetic waves and plasmonic waves in graphene is stronger than other materials.

**Figure 7:**
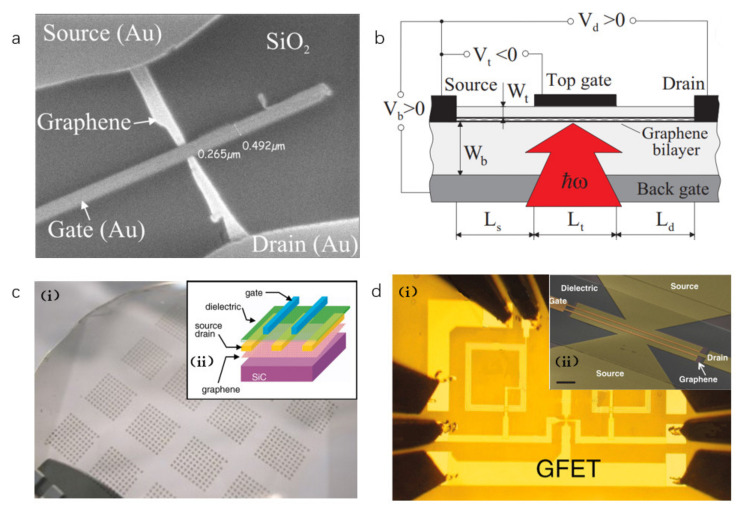
(**a**) The SEM image of a graphene device. Reprinted with permission from ref. [94]. Copyright 2021 Copyright Clearance Center. (**b**) Schematic view of a graphene terahertz radiation detector. Reprinted with permission from ref. [95]. Copyright 2021 American Physical Society. (**c**) (i) Image of devices fabricated on a 2-inch graphene wafer, (ii) the schematic cross-sectional view of a top-gated graphene FET. Reprinted with permission from ref. [96]. Copyright 2021 Copyright Clearance Center. (**d**) (i) Scanning electron image of a top-gated, dual-channel graphene transistor used in the mixer IC. (ii) Optical image of a completed graphene mixer. Reprinted with permission from ref. [97]. Copyright 2021 Copyright Clearance Center.

In 2007, Echtermeyer et al. [94] reported for the first time an FET made by graphene and suggested that graphene FET had great potential. In 2008, Ryzhii et al. developed a graphene bilayer phototransistor model and calculated its current–voltage characteristics at sufficiently large gate voltages [95]. After that, Lin et al. produced graphene-based transistors with a cutoff frequency of 100 GHz [96] and graphene-based integrated circuits (IC) [97], as shown in Figure 7. Hence, the application of the graphene field-effect transistors in the field of THz detectors has been intensively developed [71].

The working frequency of the THz detector is related to the size of the device. Therefore, to detect the higher frequency THz waves, large size graphene is needed [98,99]. Tong et al. [100] used a method that coupled small-size graphene coupled with a rectangular antenna to achieve 1–3 THz detection. In order to better adapt to the 2 THz frequency, two rectangular metal antennas are used in the article sandwich graphene to act as the source and drain of the transistor. This FET had the function of emitting and detecting THz waves. The maximum THz wave output power is 2.1 nW in the range of 1–3 THz. The external radiation is coupled to the antenna through the enhancement of the silicon lens, and the performance of the detector is significantly improved. The maximum response rate is 4.9 V/W at room temperature, and the NEP is $1.7\;\mathrm{nW}/{\mathrm{Hz}}^{1/2}$.

**Figure 8:**
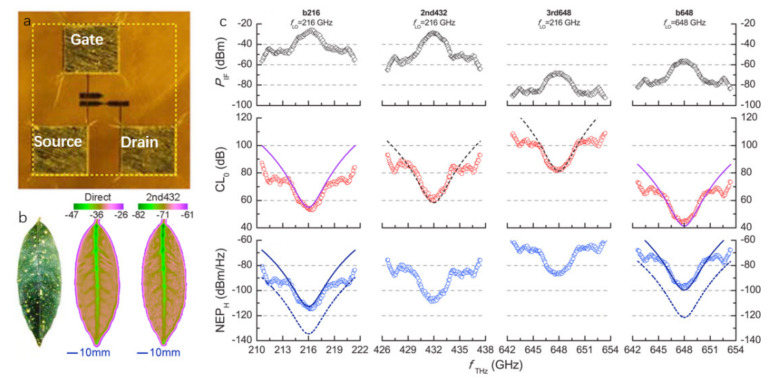
(**a**) The optical microscope image of the detector. (**b**) The photographs of the direct detection and second harmonic detection of fresh blades at 432 GHz, respectively. (**c**) The experimental results at different frequencies. The top, middle, and bottom represent the incident power, mixing loss, and noise power, respectively. Reprinted with permission from ref. [101]. Copyright 2021 Copyright Clearance Center.

Qin et al. [101] reported a GFET based on a self-mixing mechanism. The source and drain contacts to the bilayer graphene channel are two dipole antennas, as shown in Figure 8a, and the field-effect gate is located in the gap between the source and drain antennas. The THz electromagnetic wave is fed to the grid through the gate antenna, and a strong local terahertz field is induced in the graphene channel near the grid channel of the gate facing the drain antenna. The NEP of direct detection of 216 GHz and 648 GHz is $8\;\mathrm{nW}/{\mathrm{Hz}}^{1/2}$ and $1\;\mathrm{nW}/{\mathrm{Hz}}^{1/2}$, respectively. The local oscillator of 2nd harmonic (432 GHz) and 3rd harmonic (648 GHz) mixing detection of 216 GHz, and its NEP is $3\;\mathrm{nW}/{\mathrm{Hz}}^{1/2}$ and $1\;\mathrm{nW}/{\mathrm{Hz}}^{1/2}$, respectively, mixing loss is 38.4 dB and 57.9 dB, respectively. They scanned fresh leaves at different frequencies, and the results are shown in Figure 8b. Through self-mixing detection at 216 GHz, 432 GHz, and 650 GHz happened, the response characteristics of the detector are proved to meet with the design.

Mittendorff et al. [102] investigated the effect of the substrate on the detector’s performance. They used the undoped silicon substrates and heavy P doped silicon substrate coupled with metal antennas to fabricate the THz detectors. The beam is focused on the devices by off-axis parabolic mirrors, using the lock-in amplifier to measure the time-integrated photocurrent. The results illustrate that the signal rise time of the doped silicon substrate detector is about 50 ps, while the rise time of the undoped detector is within 100 ps, and the signal amplitude has also decreased. The antenna forms a capacitor on the substrate, and the substrate acts as a dielectric material, so its resistivity affects the RC time constant. In order to prove that the photocurrent generated by the detector is graphene instead of the antenna, they removed the graphene sheet on the detector and did not detect any signal under THz irradiation. The signal generated by the detector can be attributed to graphene. Under a particular wavelength range (below 20 µm), the phonons in Si absorb thermally active carriers, causing the device to heat up and the substrate resistance to decrease. Therefore, the choice of substrates using different materials (such as SiC or diamond) or different doping of silicon is also an important direction for research and improvement of graphene THz detectors.

**Figure 9:**
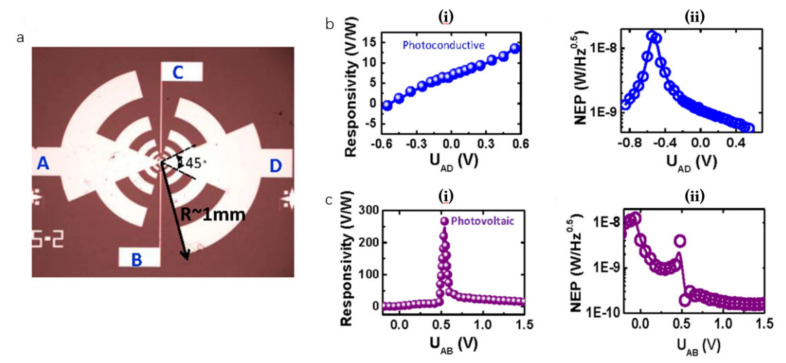
(**a**) The electrical configuration of the device, and changing the connections of the four terminals switch modes, (**b**) The responsivity and NEP curve in photoconductive mode. (**c**) The responsivity and NEP curve in photovoltaic mode. Reprinted with permission from ref. [103]. Copyright 2018 The Author.

The plasmonic wave detection requires the asymmetrical coupling of source and drain. The non-linear rectification of the carrier transmission is a direct detection, and the detection by the PTE is realized by asymmetric contact [100]. Liu et al. [103] introduced a symmetrical pair of fingers in a graphene detector based on antenna coupling, as shown in Figure 9a. There are four terminals in the detector, which can switch the light detection mode by controlling different contact configurations in the graphene channel. The function of the photocurrent can be calculated by comparing the shape of the photocurrent curve. More interestingly, the decreasing in the length of the channel or increasing the dipole interaction between the two opposite antenna arms can efficiently enhance the performance of the detector. However, by the bias field effect, the maximum voltage response of the device reaches 280 V/W. The performance of the detector is different under different working modes. As shown in Figure 9b, the NEP of the device in photovoltaic mode is $100\;\mathrm{pW}/{\mathrm{Hz}}^{1/2}$, and the responsivity is 100 V/W. When the device works in light guide mode, the performance of the device is reduced, as shown in Figure 9b. The NEP is about $500\;\mathrm{pW}/{\mathrm{Hz}}^{1/2}$, and the responsivity is about 10 V/W. There is a mismatch between the result and the theoretical result, which may be due to the super-large antenna used and the long channel have a remarkable effect on detector efficiency. It is possible that optimizing the antenna would further enhance the performance of the THz detector.

The structure parameters of the antenna also played an important role in the performance of the FET. In 2020, Viti et al. [104] encapsulated single-layer graphene (SLG) in the hexagonal boron nitride (hBN) to form a clean hBN–SLG–hBN heterostructure, which was coupled with butterfly antennas to make graphene FET. When the antenna parameters were chosen, they referred to the results of electromagnetic simulation. The radius of the antenna increased from 6 to 69 µm. At 20 µm, the in-plane component of the electric field showed a λ/2 resonance, and at 52 µm, a 3λ/2 resonance appeared. The detector uses the synchronization mechanism of photovoltaic effect and PTE to achieve low noise (NEP = $160\;\mathrm{pW}/{\mathrm{Hz}}^{1/2}$) room temperature THz detector. The responsivity is 49 V/W when exposed to 3 THz radiation at room temperature and 180 V/W at a low temperature (77 K). It can be found that changing the size of the antenna will tune its resonant frequency, and changing the antenna type can also reduce or expand its frequency coverage.

In order to increase the absorption of graphene, many effective ways such as quantum dot enhancement, antenna coupling enhancement, and plasmonic enhancement can be used [105,106,107]. Direct contact between the edge of the graphene micro-strip and the metal electrode will hinder the excitation of plasmon resonance. Mohammad et al. [108] reported on a plasmonic-enhanced THz detector. In this detector, graphene micro-strips are designed with a specific angle to make the electrode array, in which the plasmon mode is associated with currents transverse to the ribbon and can be efficiently excited by light and polarized perpendicular to the metal electrodes. They study the absorption of THz waves with different structures, including graphene micro-strips without metal electrodes, graphene micro-strips with vertical metal electrode arrays, and graphene micro-strips with inclined metal electrodes. The findings show that direct contact between the edge of the graphene micro-strip and the metal electrode prevents plasmon resonance excitation [109], and the plasmon resonance enhancement of the vertical structure is smaller than that of the graphene micro-strip without metal electrodes. Meanwhile, the enhancement in the case of the inclined structure is larger than the vertical structure enhancement. Subsequently, they shortened the distance between the electrodes to 3.8 µm, which is closer to the diffusion length of graphene hot carriers. In the same area, the short-distance structure can integrate more arrays, which would further enhance the photoelectric signal.

The interference of plasma oscillations in the channel of the field-effect transistors can be used to measure radiation polarization [110]. Sergey et al. [111] fabricated a plasmonic interferometer based on GFET. After absorbing radiation, the source and drain of the GFET excite plasma waves and interfere inside the channel. The antenna sleeves are attached to the electrodes, and the drain sleeves are bent 45 degrees to guarantee that the radiation is asymmetrically coupled to the source and drain. The amplitudes and phases of the source and drain are different, realizing a spiral-sensitive THz plasma interferometer. Changing the angle between the laser polarization and the main axes of the λ/4 could control the helicity of the radiation. They measured the helicity dependence of the photovoltage normalized by the radiation power P. The results show that when the phase of the device is asymmetrical, the helicity-sensitive response occurs, and when the signal amplitude is asymmetrical, the helicity-insensitive response arises. This work provides a basis for the phase-sensitive detection of plasma waves excited by two-dimensional materials.

The antenna will be coupled with the rectifying device by GFET, but the detector performance was affected by the rectifying of the parasitic capacitance. Gregory et al. [112] fabricated an asymmetric four-terminal ballistic rectifier structure, which relies on the ballistic motion of the carrier to rectify alternating current signals into direct current output. In the ballistic state of electron transmission, the carriers will not be randomly scattered by phonons or impurities. Electrons will be hit in a specific direction at the edge of the device to achieve a rectification effect. In this rectification method, the carriers do not need to overcome the built-in electric field, and the device threshold voltage is 0, which reduces the introduction of parasitic capacitance. They fabricated the device using graphene made by mechanical exfoliation. The responsivity is 764 V/W under 0.45 THz radiation, and the NEP is as low as $34\;\mathrm{pW}/{\mathrm{Hz}}^{1/2}$. Finally, they also achieved imaging of opaque objects under 0.685 THz radiation.

The responsivity of the traditional FET is affected by the channel conductivity, and the transconductance is limited by $ℯ/k_{B}T$. This limitation has little effect on the responsivity of the detector and is easily overlooked. Bandurin et al. [67] developed a tunnel field-effect transistor (TFET) model to rectify high-frequency signals. Since the thermoelectric plays a minor part in this scenario, the responsivity of the detector is governed by the responsivity of the channel and the tunnel junction. The tunnel barrier is essentially non-existent under normal conditions, and the responsivity is dictated by the channel responsivity. When a vertical electric field is applied to the graphene channel, a tunnel junction is formed, and the tunnel conductance is very sensitive to the junction voltage. This greatly improves the sensitivity of the TFET. In order to verify this model, a dual-gated bilayer graphene THz detector was fabricated. The responsivity of tunnel detection under 0.13 THz radiation reaches 3000 V/W, which is an order of magnitude higher than that without tunnel detection, and the NEP is about $0.2\;\mathrm{pW}/{\mathrm{Hz}}^{1/2}$. In theory, the response of this detection method can reach 100 kV/W, and after achieving the antenna and TFET impedance matching, the NEP can have a significant reduction.

## 3. Terahertz Detectors Made by Different Graphene Preparation Methods

The development of ways to manufacture graphene increases graphene application, which is an exciting occurrence. The reason is that the methods of making graphene are different, and their properties determine the application. In addition to the mechanical exfoliation method where graphene was first discovered, there are liquid exfoliation methods, molecular beam epitaxy [113], chemical vapor deposition (CVD), SiC epitaxial growth, etc. Liquid exfoliation is that exposing the graphite to organic solvents such as N-methyl-pyrrolidone. The surface tension of the solvent will cause the graphite to crack [114]. This method can make graphene on a mass scale, but the carrier mobility is small, which is suitable for transparent conductive materials or coatings [115]. Since the photon energy is only a few meV in the THz range, graphene used in THz detectors requires high electron mobility. The sensitivity of graphene THz detectors can reach the order of $\mathrm{pW}/{\mathrm{Hz}}^{1/2}$, and the response speed can reach the order of ps, but the performance of the current graphene detectors is far below the ideal value [116]. The graphene preparation methods currently used for the THz detectors mainly depend on mechanical exfoliation, CVD, SiC epitaxial growth, etc. [117].

### 3.1. Mechanical Exfoliation

Geim and Novoselov reported mechanical exfoliation for the first time in 2004 [40]. More specifically, the graphite is separated into smaller pieces, and the thinner pieces of graphite are selected. They used a specific method to divide the graphite sheet into two halves and repeated this process continuously to get thinner and thinner graphite sheets. A small number of samples are graphene composed of a single layer of carbon atoms [118,119,120]. This method of graphene manufacturing does not degrade the intrinsic characteristics of graphene, making it ideal for high-frequency electrical and optical devices.

The graphene–dielectric integration process will introduce substantial defects into pristine graphene lattices, which reduce the mobility in graphene. In addition, an immature manufacturing process leads to large access resistance. These two points lead to the poor performance of graphene THz detectors compared to other mature FET detectors [121,122,123]. Liao reported a new way to make GFET by using Co_2_Si-Al_2_O_3_ core–shell nanowires as a self-aligned top gate to fabricate graphene transistors [124]. The graphene made by mechanical exfoliation is transferred to the silicon substrate, and the Co_2_Si-Al_2_O_3_ core–shell nanowires are aligned with the graphene by the physical dry transfer method. Finally, a layer of metal is deposited on the structure. Hence, the nanowire acts as a mask, forming a self-aligned source and drain electrodes on both sides. The graphene transistor produced by this method has lower access resistance and less impurities introduced in the manufacturing process; the carrier mobility can reach 20,000 ${\mathrm{cm}}^{2}/V\cdot s$, and the cutoff frequency is 100–300 GHz.

**Figure 10:**
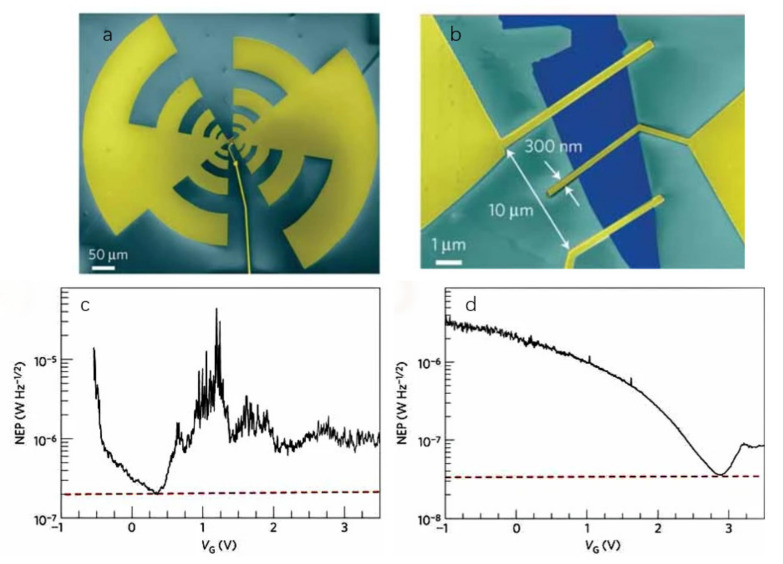
(**a**) The model of the fabricated detector. (**b**) The channel length is 10 μm, with a gate width 300 nm. (**c**) The curve of NEP with single-layer graphene, and (**d**) The bilayer graphene in which the dotted line indicates the minimum value. Reprinted with permission from ref. [122]. Copyright 2021 Copyright Clearance Center.

Vicarelli et al. [122] used single-layer and bilayer graphene prepared by mechanical exfoliation to fabricate an antenna-coupled graphene FET. The coupled antenna is a log-periodic antenna, as shown in Figure 10a, and the channel length is demonstrated in Figure 10b, which is a low parallel capacitance antenna that leads to ensure the selectivity of the spatial mode and the polarization of the incident radiation. Furthermore, the antenna can also ensure the asymmetry of the source and drain. The plasma wave excited in the channel of the FET realizes the detection of 0.3 THz at room temperature. As shown in Figure 10b,c, the NEP and responsivity of the single-layer graphene detector are $200\;\mathrm{nW}/{\mathrm{Hz}}^{1/2}$ and 0.1 V/W, and the NEP and responsivity of the bilayer graphene detector are $30\;\mathrm{nW}/{\mathrm{Hz}}^{1/2}$ and 0.15 V/W, respectively.

Packaging graphene with standard dry skin technology can provide the cleanest environment for graphene [125]. Bandurin et al. [126] using graphene made by mechanical exfoliation and encapsulated by hBN. The carrier mobility is as high as 30,000 ${\mathrm{cm}}^{2}/V\cdot s$ at room temperature. They fabricated several devices of different sizes and used the gate to tune plasmon speed to conduct experiments in multiple resonance modes. FET devices with a feature size of 4 µm have observed plasmon resonance in the frequency range of 0.13–2 THz. At 0.13 THz, the NEP is as low as $10^{-13}\;W/{\mathrm{Hz}}^{1/2}$, and the responsivity is 3000 V/W (10 K). The performance is comparable to bolometers (commercial devices) operating at low temperatures (below 5 K). It indicates that a high-mobility graphene FET that uses far-field coupling to incident radiation can be used as a resonant THz photodetector, and it demonstrates the remarkable advantages of graphene materials with high mobility in THz detection.

### 3.2. Chemical Vapor Deposition

Under high temperatures and certain conditions, carbon atoms are deposited on the metal surface using a mixture of methane and hydrogen or other carbon sources to form large-area graphene film [127,128]. Chemical vapor deposition mainly includes thermal CVD, plasma-enhanced CVD, and hot filament CVD [129,130,131]. These methods can control the growth thickness and size of graphene. At the same time, it can achieve uniform doping of graphene by introducing heterogeneous materials such as nitrogen and carbon, which is beneficial to the functionalization or fine-tuning of the graphene transistors [132]. The carrier mobility of the graphene prepared by the CVD method can reach 40,000 ${\mathrm{cm}}^{2}/V\cdot s$ at 300 K and 180,000 ${\mathrm{cm}}^{2}/V\cdot s$ at 2 K [133]. This CVD-grown graphene has low cost and high quality, which makes it one of the most widely used methods.

Zak et al. explored the use of CVD-grown graphene for the detection of THz signals [134]. They transfered the graphene grown on copper foil to the substrate using a frame-assisted bubbling transfer process. The mobility of electrons and holes in the fabricated device is 1800 ${\mathrm{cm}}^{2}/V\cdot s$ and 1200 ${\mathrm{cm}}^{2}/V\cdot s$, respectively. This detector is coupled with a slit-structured butterfly antenna. The resistance is calculated by the ratio of ΔU and ΔI of the rectified voltage, and the current response is measured under THz self-mixing. This calculation result is consistent with the direct current and voltage measurement calculation results, ensuring that the measurement result is perfect and reliable. The device can detect THz signals at 0.6 THz at room temperature, and the measured maximum responsivity and minimum NEP are 14 V/W and $\;515\;\mathrm{pW}/{\mathrm{Hz}}^{1/2}$, respectively. This work proves that CVD graphene-based THz detectors have great potential.

**Figure 11:**
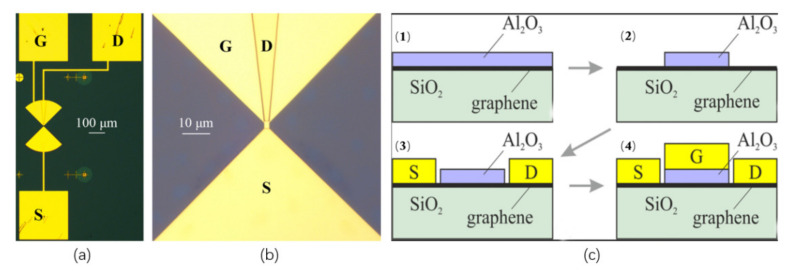
(**a**,**b**) The optical microscope image of the fabricated GFET detector (S, G, and D—source, gate, and drain, respectively) and the image of the GFET detector in the center of the antenna. (**c**) Schematic illustration of the fabrication steps of the graphene FET detector. (1) The graphene layer was covered by a thin Al seed layer and followed by natural oxidation; then, an Al_2_O_3_ gate dielectric was grown on top of the seed layer by atomic layer deposition. (2) The Al2O3 was patterned and etched with a buffered oxide etch. (3) The source and drain contacts were fabricated by evaporating. (4) The gate electrode was evaporated and patterned on top of the patterned Al_2_O_3_ layer. Reprinted with permission from ref. [135]. Copyright 2021 Copyright Clearance Center.

However, the fabrication process of the device will introduce impurities in the graphene, which will affect the performance of the detector. In order to solve this problem, Generalov et al. [135] used CVD-grown graphene to design and fabricate a special room-temperature graphene-based THz detector with a working frequency of 400 GHz. The detector has a slit butterfly-shaped asymmetric antenna structure with a radius of 160 µm, as shown in Figure 11a,b. They improved the device manufacturing process on Zak’s work [134]. We used the pre-growing dielectric gate method to deposit a layer of dielectric Al_2_O_3_ gate and then make the source and drain, as shown in Figure 11c, which ensures that other substances do not touch the graphene in the FET channel in the subsequent measures. The signal from the antenna is fed to the gate-source terminal. The gate-drain terminal has capacitive coupling, which is achieved by separating the bow-tie antenna. The electron and hole mobility of the device made by this method are 3100${\;\mathrm{cm}}^{2}/V\cdot s$ and 2800 ${\mathrm{cm}}^{2}/V\cdot s$, respectively. The maximum responsivity reaches 74 V/W, and the NEP reaches $\;130\;\mathrm{pW}/{\mathrm{Hz}}^{1/2}$.

### 3.3. SiC Epitaxial Growth

A graphene layer can be formed on the silicon or carbon surface of the SiC wafer by sublimating the Si atoms on the surface of the SiC (0001) wafer and annealing the remaining C atoms to graphitize them [136,137]. Without the need for further transfer, this process can prepare large-scale and high-quality wafer-level graphene. Its electrical properties are similar to those of single-layer graphene produced by mechanical exfoliation, with mobility of up to 6600 ${\mathrm{cm}}^{2}/V\cdot s$ [138], which can be directly applied to the development of electronic devices.

An FET made of SiC epitaxial growth graphene was reported by Bianco [139]. The detector incorporates two mechanisms for the detection of THz. One is plasmonic detection due to the non-linearity of electron transport, and the other is the thermoelectric effect due to the presence of junctions of carrier density and induced temperature gradient through the FET. They investigate the contribution of the relevant processes using the particular dependency of positive gate voltages. The results suggest that plasmonic detection plays a vital role in the detection, but it is decreased by the thermoelectric impact. At the frequency of 295 GHz and 353 GHz, the responsivity is 0.25 V/W, 0.15 V/W, and NEP is $80\;\mathrm{nW}/{\mathrm{Hz}}^{1/2}$, $160\;\mathrm{nW}/{\mathrm{Hz}}^{1/2}$, respectively.

**Figure 12:**
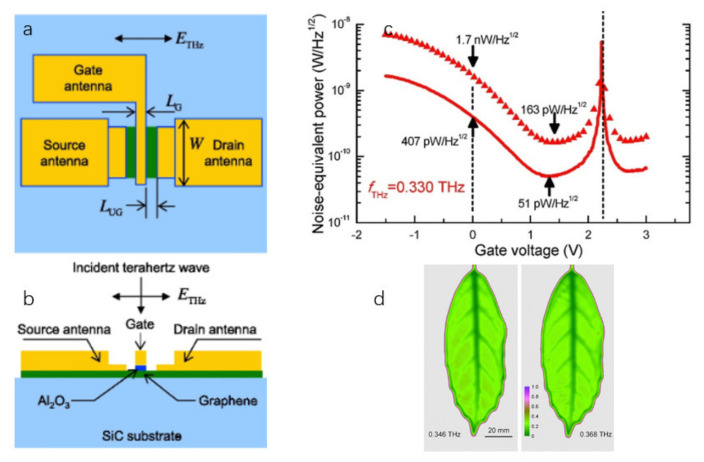
(**a**,**b**) The top view and front view illustration of the fabricated detector, respectively. (**c**) The red curve calculated from the responsivity at 0.330 THz and the thermal noise of the detector, and the triangle line is calculated based on the responsivity and the noise spectral density dominated by the voltage pre-amplifier. (**d**) The photo of using this detector to image fresh leaves. Reprinted with permission from ref. [140]. Copyright 2021 Copyright Clearance Center.

Qin et al. [140] fabricated an FET based on bilayer graphene. A 2-inch 4H-SiC (0001) graphene wafer was used as substrate-grown monolayer graphene at a high temperature of 1550 °C in an argon atmosphere. Then, they annealed the monolayer epitaxial graphene in molecular hydrogen at 900 °C to obtain the bilayer graphene. The structure of the detector is shown in Figure 12a,b. The detector has a voltage responsivity of 30 V/W and a NEP of $51\;\mathrm{pW}/{\mathrm{Hz}}^{1/2}$ at the 0.33 THz. Transmission-type THz imaging of a fresh leaf was studied by using the GFET detector with the gate floating as a proof-of-concept experiment on a two-terminal detector. As shown in Figure 12d, a GFET detector with a floating gate was used to study the transmission-type THz imaging of fresh leaves.

Rama et al. fabricated a THz detector coupled with a split-bow-tie antenna on a 4H-SiC (0001) substrate [141]. The photovoltage of this device can be written as $\Delta U=\Delta U_{\mathrm{pwg}}+\Delta U_{\mathrm{pte}}$, where $\Delta U_{\mathrm{pwg}}$ is the voltage generated by the plasma effect, and its magnitude is determined by the conductivity and coupling efficiency of incident radiation to the antenna. The thermal effect generates a voltage, $\Delta U_{\mathrm{pte}}$, whose magnitude is proportional to the conductivity. The change in conductivity is attributed to the change in carrier density. In order to obtain a large photovoltage, it is essential to choose graphene with high carrier mobility. However, the plasma effect plays a major role in the detection process. So, the energy coupled to the antenna is the most important in the design. By enhancing the antenna coupling efficiency, their device has a responsivity of 535 V/W at a frequency of 0.8 THz, and the NEP is less than $100\;\mathrm{pW}/{\mathrm{Hz}}^{1/2}$.

The resistance of the epitaxial growth graphene on SiC varies greatly with temperature due to quantum containment, so it has a high response rate [136,142]. Abdel El Fatimy et al. [107] used semi-insulating 6H-SiC to synthesize graphene and made thermionic detectors by electron beam lithography. The detector performance is based on Joule heating. The responsivity of the detector is estimated according to the relationship diagram between resistance and electric power, which can reach $0.65\times 10^{10}\;V/W$ at 6 K. Then, using the reverse wave oscillator on an incident 0.15 THz electromagnetic wave, the detector has a response at $5\times 10^{10}\;V/W$, and the NEP is $2\times 10^{-16}\;W/{\mathrm{Hz}}^{1/2}\;\left(2.5\;K\right)$. This noise power is evaluated based on the electrical characteristics and absorbed power of the pyrometer (${\mathrm{NEP}}^{2}={\;\mathrm{NEP}}_{\mathrm{JN}}^{2}+{\;\mathrm{NEP}}_{\mathrm{SN}}^{2}+{\;\mathrm{NEP}}_{\mathrm{TF}}^{2}$, JN is Johnson–Nyquist noise, SN is hot noise, and TF is thermal fluctuations noise). Compared with the optical NEP measured by the incident power, the electrical NEP can directly reflect the intrinsic performance of the device without considering the antenna and measurement circuit. Using the detector coupled with a log-periodic antenna, the working range is increased to 0.7–4 THz. However, it is not clear whether the performance of the quantum dot detector is related to the higher frequency. In their subsequent work, the different wavelengths (from THz to ultraviolet light) are used to measured responsivity and calculate the total NEP, and the results show that the performance of the detector is completely independent of photon energy and the specific wavelength [143].

## 4. Challenges and Future Perspective of Graphene Based Detectors

Although many scholars have made progress on graphene THz detectors, there are still great challenges. For the preparation of graphene, the methods mentioned above have their limitations. The graphene produced by mechanical exfoliation has high quality, but the process is hard to control, and the produced graphene can hardly be mass-produced (with different areas and different layers), while large-area graphene also can hardly be produced. These limitations make it difficult to make the graphene THz detectors. The graphene synthesized by CVD can be controlled by changing the carbon source gas, deposition time, temperature, and other factors to control the size and layers of graphene [144]. Although the graphene produced by CVD has high quality, the synthesized graphene is attached to the surface of the metal substrates. They still need to be transferred to the insulating substrates before making the detector. During the transfer process, graphene could easily be contaminated or fragile, which remarkably influences the properties of graphene materials [145]. The most commonly used is the polymer-supported transfer method, which can completely transfer graphene to various substrates, but the etching solution and residual polymer contamination during the transfer process will damage the quality of the graphene and harmful to their mobility [146]. SiC epitaxial growth graphene can also control the size and number of layers. Comparing with CVD growth graphene, it is directly located on the SiC substrate and does not need a transfer. However, there is no dielectric isolation between the graphene and the substrate, and the charge of the substrate will affect the performance of the detector [147]. In addition, the harsh growth environment, high substrate price, low growth efficiency, and other factors make this method also difficult to achieve mass production in the near future.

There are also many challenges in the making of the graphene THz detector. For example, the detector is usually coupled with a metal antenna, but this will inevitably introduce some parasitic capacitance and increase the access resistance. When designing a specific antenna, scientists not only need to consider the size and type of the antenna but also need to take care of the impedance matching problem. Impedance mismatch will increase the NEP and RC time constants of the detection circuit [148]. When preparing the electrode, the material of the metal and the thickness of the electrode would also introduce impedance mismatch. Moreover, the contact interface between the metal and the graphene will also cause a step barrier due to their different work function, and the connection resistance in this area is also relatively high. In this case, it is not only necessary to avoid contamination and material degradation during the fabrication process, but it is also necessary to select the appropriate metal and thickness in order to lower the electrode’s connection resistance [149]. In the bolometer, in order to improve the sensitivity, it is very necessary to choose a metal with small thermal resistance. The carrier mobility of graphene is limited by the substrate; choosing a high-quality substrate to fabricate the detector will reduce the influence of charged surface states and impurities, surface optical phonons, and substrate surface roughness [150]. Using Al_2_O_3_ or diamond instead of SiO_2_ as the substrate or encapsulating graphene with hBN may be employed in the future to develop GFET [151]. In addition, a standard process of electron beam lithography, dielectric layer deposition, graphene metal connection, and other steps in the device preparation process also need to be developed for making highly reliable graphene detectors.

Despite the challenges, there has been continuous progress in this field. CVD can prepare graphene materials with a large area and consistent performance with a single crystal and can use the direct transfer layer method to achieve clean and non-destructive transfer of graphene [152]. In addition, new structures of THz detectors have been continuously proposed [103,112], which can meet the needs in different environments. The imaging system using the graphene THz detector can realize high-definition imaging, and the graphene FET can realize high-speed communication. They will play an important role in medical treatment, anti-terrorism, communications, and astronomical exploration in the future.

## 5. Discussion and Conclusions

In this review, we introduce the recent developments in graphene THz detectors. The review discussed the methods reported by researchers to improve the performance of THz detectors, which are based on graphene made by different methods. The performances of the detectors mentioned in the article are shown in Table 2.

Although the graphene THz detector has made a lot of progress in the laboratory, and they show many promising prospects comparing to traditional detectors, there is still a long way to go to achieve commercialization application. Moreover, the working range of these THz detectors is mostly at low-frequency THz waves, and high-frequency THz detectors are still difficult to achieve. These THz detectors with low equivalent noise power and high responsivity need to be realized in a low-temperature environment, and room temperature detectors still need to be further studied.

## Figures and Tables

**Table 2:** Performance parameters of graphene terahertz detectors mentioned in the article.

Mechanism	Graphene Manufacturing Method	Working Frequency	Working Temperature	NEP	Responsivity	Reference
Bolometer	SiC epitaxial growth	0.3–1.6 THz	3 K	$5.6\;\mathrm{pW}/{\mathrm{Hz}}^{1/2}$	-	[66]
	SiC epitaxial growth	0.7–4 THz	2.5 K	$0.2\;\mathrm{fW}/{\mathrm{Hz}}^{1/2}$	$10^{10}\;V/W$	[107]
PV	CVD	2 THz	RT	$150\;\mathrm{nW}/{\mathrm{Hz}}^{1/2}$	34 μA/W	[73]
PTE	mechanical exfoliation	1 THz	RT	$100\;\mathrm{pW}/{\mathrm{Hz}}^{1/2}$	10 V/W	[74]
	CVD	0.11–0.3 THz	RT	$0.35\;\mathrm{nW}/{\mathrm{Hz}}^{1/2}$	28 V/W	[75]
	mechanical exfoliation	1.8~4.2 THz	RT	$80\;\mathrm{pW}/{\mathrm{Hz}}^{1/2}$	105 V/W	[72]
Plasma wave resonance	mechanical exfoliation	0.3 THz	RT	$30\;\mathrm{nW}/{\mathrm{Hz}}^{1/2}$	0.15 V/W	[122]
	SiC epitaxial growth	0.3–0.35 THz	RT	$80\;\mathrm{nW}/{\mathrm{Hz}}^{1/2}$	0.25 V/W	[139]
	SiC epitaxial growth	0.2–0.65 THz	RT	$1\;\mathrm{nW}/{\mathrm{Hz}}^{1/2}$	2.65 V/W	[101]
	mechanical exfoliation	1–3 THz	RT	$1.7\;\mathrm{nW}/{\mathrm{Hz}}^{1/2}$	4.9 V/W	[100]
	CVD	0.6 THz	RT	$515\;\mathrm{pW}/{\mathrm{Hz}}^{1/2}$	14 V/W	[134]
	SiC epitaxial growth	0.34 THz	RT	$51\;\mathrm{pW}/{\mathrm{Hz}}^{1/2}$	30 V/W	[140]
	CVD	0.35 THz	RT	$130\;\mathrm{pW}/{\mathrm{Hz}}^{1/2}$	74 V/W	[135]
	-	3 THz	77 K	$160\;\mathrm{pW}/{\mathrm{Hz}}^{1/2}$	180 V/W	[104]
	CVD	0.1–0.3 THz	RT	$100\;\mathrm{pW}/{\mathrm{Hz}}^{1/2}$	280 V/W	[103]
	mechanical exfoliation	0.07–0.69 THz	RT	$34\;\mathrm{pW}/{\mathrm{Hz}}^{1/2}$	764 V/W	[112]
	mechanical exfoliation	0.13 THz	10 K	$0.2\;\mathrm{pW}/{\mathrm{Hz}}^{1/2}$	3000 V/W	[67]
	mechanical exfoliation	0.13–2 THz	10 K	$0.1\;\mathrm{pW}/{\mathrm{Hz}}^{1/2}$	3000 V/W	[126]
